# The Effect of Periodontal Treatment on Clinical and Biological Indicators, Quality of Life, and Oral Health in Rheumatoid Arthritis Patients: A Quasi-Experimental Study

**DOI:** 10.3390/ijerph19031789

**Published:** 2022-02-04

**Authors:** Adriana Posada-López, Javier Enrique Botero, Ricardo Antonio Pineda-Tamayo, Andrés A. Agudelo-Suárez

**Affiliations:** 1 Faculty of Dentistry, University of Antioquia, Medellín 050010, Colombia; adriana.posada@udea.edu.co (A.P.-L.); javier.botero@udea.edu.co (J.E.B.); 2 Clinical Information Group, Artmedica Institution (IPS), Medellín 050030, Colombia; ricardopineda@artmedica.com.co

**Keywords:** rheumatoid arthritis, periodontitis, oral health, quality of life, periodontal diseases/therapy

## Abstract

Non-surgical periodontal therapy (NSPT) has been shown to have systemic effects. It has been suggested that, similar to rheumatoid arthritis (RA), periodontitis (PD) has an impact on general health, in terms of psychological, physical, and social aspects. This study determines the effect of periodontal treatment in RA activity, health-related quality of life, and oral health self-perception before and after periodontal treatment in RA patients. A quasi-experimental, prospective, non-randomized study was conducted, and 52 patients were included in the study. Periodontal parameters and the instruments disease activity score-28 (DAS-28), SF-36, and OHIP-14 were measured at baseline and at 3 months after NSPT. All differences were statistically assessed. The study protocol was registered in Clinical Trials (NCT04658615). No statistically significant differences were found in the scores of DAS-28 before and after the intervention in the group with PD and reduced periodontium. When the effect of periodontal treatment was analyzed in the group of 29 patients who were followed up, it was found that there were statistically significant differences before and after in variables such as psychological distress, emotional role, and mental health, which indicates an improvement in the scores of these variables. NSPT influenced the health-related quality of life measured with SF-36 and OHIP-14 in patients with RA. In conclusion, NSPT has an effect on self-reported quality of life and health indicators more than the RA activity as measured with DAS-28. However, the clinical effect of periodontal treatment in RA patients provides important data to support periodontal care in patients.

## 1. Introduction

Periodontitis is a common oral disease related to chronic degenerative changes, such as variation among tissues, physiological and immunological changes, etc. [1]. Periodontitis is characterized by pathological loss of periodontal ligament and alveolar bone and involves complex dynamic interactions between active microorganisms, specific bacterial pathogens, and destructive immune responses [2]. Periodontitis prevalence in the world varies between 20% and 50% [3], and in Colombia, according to the country’s fourth National Oral Health Study, it is 73% [4].

On the other hand, rheumatoid arthritis (RA) is an immune system disease that causes cartilage damage and joint instability, leading to degenerative changes and functional loss to the point of causing an inability to move [5]. Its prevalence in the world population varies between 0.2% and 5.0%, and around 1% in Colombia [6]. It is more frequent in women than in men, with a ratio of 4:1, and it increases with age [6]. As for possible causal factors, they include environmental, genetic, and infectious ones; nevertheless, research on other possible RA causes is still underway, which aims to clarify and understand the pathogenic mechanisms of the disease, as well as its possible clinical, diagnostic, and treatment applications [7].

Both RA and periodontitis are chronic diseases that cause connective tissue and bone destruction [8]. These two diseases are currently considered to share etiopathological mechanisms and are, therefore, said to be related [8]. Recently periodontitis has been related to RA as they share immunopathological mechanisms relevant to the inflammatory process leading to connective tissue and bone destruction [9,10].

Scientific evidence about the RA–periodontitis association mentions an epidemiological relationship, even with the methodological limitations these studies present [11,12]. By contrast, other studies suggest a clinical correlation arising from etiopathogenesis [13], and other studies refer to the possibility of a reciprocal relationship [14,15]. It is not clear whether RA is a risk factor for periodontitis or vice versa, but a higher incidence and prevalence of periodontal disease and tooth loss have been found in RA patients, compared with healthy individuals [15].

Regarding the correlation with blood markers, some studies show a lack of correlation between anti-citrullinated peptide antibodies (ACPAs) and periodontitis clinical parameters, suggesting that the citrullination of proteins during periodontitis pathogenesis may not be sufficient to establish a causal link between RA and periodontitis [16]. Another study concluded that citrulline levels in saliva are not associated with the severity of RA disease, despite significant differences in their periodontal status [17]. Other studies link these diseases since the treatment stage with specific medications for RA, as well as with the way in which these are associated with some periodontal health parameters, and how they can affect the periodontal health of this group of patients [18]. Moreover, they indicate that non-surgical periodontal therapy has a beneficial effect on the signs and symptoms of RA, regardless of the medications used to treat this condition [19]. Some research has focused on studying the effectiveness of non-surgical periodontal therapy from a medical viewpoint, in which blood and pharmacological parameters related to the presence or absence of the disease are measured [10,20]. This leads to the conclusion that untreated periodontitis results in tooth loss with a negative impact on oral-health-related quality of life (OHRQoL) [21]. Its impact on general health has been proven in various studies [22].

RA has been studied in the context of the burden that the disease represents for families and for society since the disease is classified as high cost, as well as due to the impact it has socially and at work. This is particularly true, as it generates disability, an aspect that affects and significantly deteriorates the patient’s health-related quality of life (HRQoL) [23]. RA is one of the most disabling autoimmune diseases and commonly involves pain; most people enduring the disease have a mild-to-moderate disability, and in less than 10%, it is classified as severe [24].

Due to its chronic nature, RA is classified as high cost, as it causes disability in its more advanced stages [25]. Evidence suggests that chronic diseases generate an economic burden given the complex and expensive treatments needed for long periods of time [26]. It has been suggested that, similar to RA, periodontitis has an effect on general health, in terms of psychological, physical, and social aspects [27].

Accordingly, the aim of this study was to determine the effect of periodontal treatment in RA activity, health-related quality of life, and oral health self-perception before and after periodontal treatment in RA patients.

## 2. Materials and Methods

### 2.1. Design, Population, and Sample

A quasi-experimental, prospective, non-randomized study was conducted. The information was gathered from a primary source, and each patient served as his or her own control (pretest–posttest). This particular type of study was conducted because randomization of patients to treatment allocation was not possible, on the grounds that every patient who is diagnosed with periodontal disease must be treated for ethical reasons.

**Figure 1:**
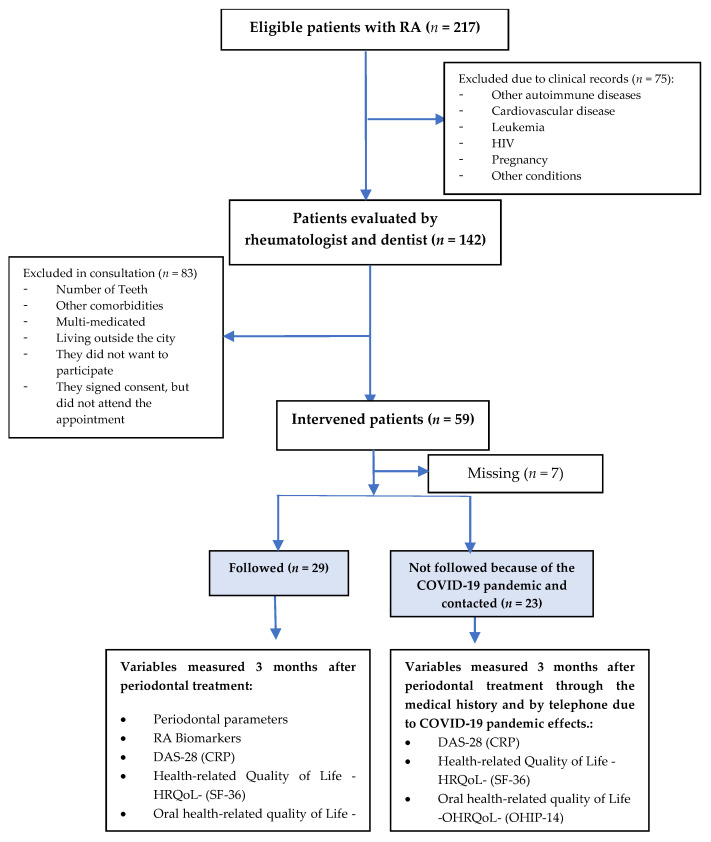
Patients’ recruitment flow diagram.

**Figure 2:**
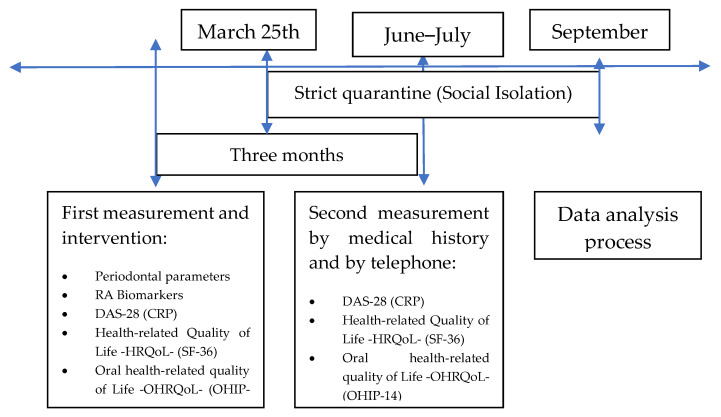
Data collection behavior due to the COVID-19 pandemic during 2020.

The study population was made up of patients who consulted a specialized clinic in Medellín, Colombia, and were diagnosed with seropositive RA. In total, 52 patients were included in the study. No sampling procedure was performed, and information was collected from those who met the selection criteria (inclusion and exclusion)—first, by reviewing medical records as initial medical criteria and comorbidities filter and then by consulting with the treating physician and verifying the selection criteria with a checklist. According to the monitoring and measurement of variables, two groups were defined: one with both measurements (before and after) of all the variables, and another for whom, due to SARS-CoV-2 pandemic confinement, HRQoL and OHRQoL variables could only be measured by telephone and DAS-28 by means of the treating physician who noted it in their clinical record (Figure 1 and Figure 2).

### 2.2. Selection Criteria

Individuals were included if they met the following criteria: ≥18 years; confirmed RA diagnosis according to the American College of Rheumatology [28] with a disease activity score-28 (DAS-28-CRP) ≥ 3.2 and no changes in RA medication in the previous 3 months; at least 15 teeth excluding third molars. Non-RA participants met the same inclusion criteria except for the RA parameters and confirmed diagnosis. On the contrary, possible participants were excluded when they had periodontal treatment or antibiotics use in the previous 3 months; uncontrolled diabetes; HIV; liver disease; head and neck radiation therapy; pregnancy; use of cyclosporine. Smoking, hypertension, and hyperlipidemia were not considered exclusion criteria and were recorded accordingly for analysis.

### 2.3. Data Collection Techniques

#### 2.3.1. Physical and Periodontal Examinations

A single experienced clinician performed the complete periodontal examination using a periodontal probe (UNC-15) and recorded the gingival margin, probing depth (PD), periodontal attachment level (PAL), and bleeding on probing (BOP) in six sites per tooth excluding third molars. Periodontitis was defined as 2 or more non-adjacent interdental sites with loss of PAL, PD ≥ 4 mm, and BOP. The periodontitis stage (stage I–IV) was determined according to the current classification of periodontal diseases [29]. Cases of clinical gingival health or gingivitis in a reduced periodontium in patients with stable periodontitis or without periodontitis were characterized by attachment level loss (PAL), PD ≤ 3 mm, and BOP [30]. Moreover, the periodontal inflammatory burden index (PIBI) was calculated by adding the number of sites with moderate periodontitis (4–5.9 mm) to the weighted number of sites with severe periodontitis (≥6 mm) in the PIBI formula = ∑ (NmodPD + 2 NadvPD) [31]. The clinical researcher recording periodontal parameters was calibrated for repeated measurements prior to patient inclusion (Kappa value was ≥ 0.80 for PAL and PD).

Subgingival plaque samples were taken from the 5 deepest sites using sterile paper points inserted into the bottom of the sulcus or pocket for 30 s and were then pooled in a vial containing viability medium Göteborg anaerobically (VMGA) III transport medium. All samples were processed within 24 h and incubated in anaerobic culture chambers using Brucella Blood Agar for the detection of *Porphyromonas gingivalis*.

Levels of anti-citrullinated protein antibodies (ACPAs; U/mL), rheumatoid factor (RF; U/mL), and high sensitivity C-reactive protein (CRP; mg/L) were measured in peripheral blood samples from all participants in a reference laboratory using standardized laboratory methods.

RA patients were examined by a trained rheumatologist during the study and the rheumatologist confirmed the diagnosis. Medical information was obtained from their medical records, including RA duration, (DAS-28-CPR) and current medication for RA (non-steroidal anti-inflammatory drugs (NSAIDs), biological and non-biological disease-modifying antirheumatic drugs (DMARDs), and corticosteroids). Non-biological DMARDs included hydroxychloroquine, methotrexate, sulfasalazine, and leflunomide. Biological DMARDs included adalimumab, etanercept, abatacept, golimumab, infliximab, rituximab, and tocilizumab.

#### 2.3.2. Periodontal Procedures

Non-surgical periodontal therapy (NSPT) was administered within 5 days of inclusion. A single 1 h session of full-mouth debridement (supragingival and subgingival) was performed by an experienced clinician, with an ultrasonic device and Gracey curettes, under local anesthesia. The intensity of the NSPT was clinically determined by the clinician according to the characteristics of each case (probing depths, bleeding on probing, and recession). Therefore, patients with reduced periodontium and gingival inflammation received NSPT with less intensity than patients with periodontitis. Upon completion of the NSPT, all surfaces were polished using rubber cups and prophylaxis paste, and each patient received oral hygiene instructions and an oral care package that included a toothbrush and toothpaste (Vitis Encias Medium toothbrush; Vitis Encias toothpaste; Dentaid, Colombia). No additional oral hygiene measurements such as chlorhexidine mouthwash were administered during the study period. We decided to use this type of single 1 h session based on patients’ facilities, therapy costs, and objectives of this study.

It is important to note that the periodontal intervention was performed in all patients because it is aimed at biofilm control, which is why patients without periodontitis still received periodontal therapy; this is recommended for all individuals to maintain their periodontal health. Additionally, it was an interesting comparison group since it allowed observing the effect of therapy alone in the absence of deep signs of periodontal inflammation. Furthermore, since it was not RCT, it was unethical to leave them without therapy.

### 2.4. Variables

The primary outcome result was the change in DAS-28 values. The “disease activity score” (DAS) is a scoring system for the assessment of RA activity [32]. This system is recommended for clinical studies and for daily clinical practice. DAS stands for “disease activity score”, and the number 28 refers to the 28 joints that are examined in this assessment: (1) count the number of swollen joints (out of the 28); (2) count the number of tender joints (out of the 28); (3) C-reactive protein (CRP), patient global disease activity (PGDA—patient’s self-assessment of overall RA disease activity on a scale 1–10, where 10 is maximal activity). A complex mathematical formula is applied, and the disease activity is classified in remission <2.6, low 2.6–3.2, moderate 3.2–5.1, and high >5.1.

Changes in HRQoL and OHRQoL were included as secondary outcomes. SF-36 is considered a validated HRQoL tool that can be used in several medical conditions. It has been translated and validated in many languages and cultures, including Medellín, Colombia [33]. SF-36 contains eight different dimensions of HRQoL: physical functioning, social functioning, role limitations due to physical functioning (role functioning—physical), bodily pain, general mental health, role limitations due to emotional functioning (role functioning—emotional), vitality (energy and fatigue), and general health perception. For additional information about the scores and their mathematical formula, the original design of this instrument could be consulted [34]. As for the administration, it says that it must be self-completed, by direct interview and by telephone, and it takes between 5 to 10 min to be answered [35].

For OHRQoL, the OHIP-14 tool was used. The OHIP-14 is a shortened version of a scale and includes 14 questions to assess seven dimensions of the impact of oral conditions on people’s quality of life: functional limitation, physical pain, psychological discomfort, physical disability, psychological disability, social disability, and handicap. Responses are recorded on a five-point ordinal scale coded 0 = never, 1 = hardly ever, 2 = occasionally, 3 = fairly often, and 4 = very often. The questionnaire is available in Spanish, and it has been validated for use in epidemiological studies [36]. Data provided for the questions of the OHIP-14 were used to calculate three summary indicators, as used previously in the literature [37]: (1) Prevalence= the percentage of respondents reporting one or more impacts “fairly often” or “very often”. This variable identifies those whose oral health impacts are chronic rather than transitory; (2) Extent: the number of items reported “fairly often” or “very often”; (3) Severity: the sum of the response codes for the 14 items. This takes into account impacts experienced at all levels of frequency. Given the response codes, severity scores range from 0 to 56, with higher values indicating more frequent impacts. Additionally, for each dimension, the response codes were summed, with a range from 0 to 8. Higher impacts in the three summary variables are related to overall lower OHRQoL. Such measures have been used in other research at the international level [38] and in the city of Medellín [39].

All variables were measured as the difference between the initial examination and the one made 3 months later. It was carried out in two moments: the first moment or pretest, in which the HRQoL and OHRQoL, as well as the RA-related clinical and periodontal variables. After the first moment, the procedure was performed, which for this study was non-surgical periodontal therapy. The second moment, or posttest, was carried out 3 months after the procedure (Figure 1 and Figure 2).

Other variables included the following three groups: sociodemographic, RA clinics, and periodontitis.

### 2.5. Statistical Analysis

Sample size calculation was based on the detection of a 0.6-point change in DAS-28-CRP. This value (0.6) was chosen since it is the minimum therapeutic response according to the European Alliance of Associations for Rheumatology (EULAR) [40]. A standard deviation of 0.6 [41] and a two-tailed test, at a significance level of 5%, were assumed to obtain a study test power greater than 80%.

First, for HRQoL, the scores for each SF-36 dimension were calculated, where FF represents physical function; RF, physical role; RE, emotional role; FS, social function; DC, corporal pain; V, vitality; SM, mental health. The scores for each of these were obtained using the following expressions: FF = (SF-10/20), RF = (SF-4)/4, RE = (SF-3)/3, FS = (SF-2)/8, DC = (SF-2)/10, V = (SF-4)/20, and SM = (SF-5)/25. Once the assignments of the previous scores had been made, the general health score, SG = (SF-5)/20, was calculated, where SF is the sum of the scores obtained with the questions of that domain. Afterward, the normality of the scores obtained for each of the dimensions was validated in the pretest for HRQoL. Similarly, the oral health self-perception domains were assessed using the Shapiro–Wilk test. For population averages, means, and their respective interquartile ranges (IQRs), 95% confidence intervals were established. The Posttest procedure was analogous, and then, pretest and posttest measurements were compared, the assumption of normality having been tested.

Effect sizes were calculated using the following expression [42]: $d=c\left(n-1\right)\times \frac{{\bar{y}}_{pre}-{\bar{y}}_{post}}{S_{pre}}$, where ${\bar{y}}_{pre}$ and ${\bar{y}}_{post}$ are the scores averages in the pretest and posttest, respectively, and $S_{pre}$ is the standard deviation in the pretest; c(n−1) is the correction factor with c(n−1)=1−3(4×n−5). As the scores did not comply with the normality assumption, the non-parametric Wilcoxon test was used to assess the differences in average ranges, accompanied by the non-parametric effect size using the following expression [41]: $d=\frac{Q_{post}-Q_{pre}}{S_{bip}}$, where Qpost and Qpre are posttest and pretest medians in, respectively, and Sbip is the two-weighted standard deviation of the control group for each dimension in the pretest.

Effect size values were taken as absolute values, and the interpretation of them followed the proposals of Cohen et al. [43] and Grissom et al. [41]. The McNemar test was also used to assess the significance of changes in HRQoL and self-perception in health categories before and after treatment. For multivariate analysis, according to the volume of variables under analysis, a factor analysis with principal component analysis (PCA) was carried out as input for establishing a multiple linear regression model in which the effect of nonsurgical periodontal therapy and confounding factors, as well as variables that were biologically plausible, was evaluated.

Criteria for performing the PCA were as follows: sample size of 50 subjects, compliance with quantitative variables interdependence criteria, and correlations greater than 0.5, whose determinant value was 0.00. The chosen rotation method was Varimax, and those variables with extraction values below 0.75 were omitted, given that the sample size was 52 subjects. For extracting the main components, those that explained 60% or more of the variance and the subsequent verification of the inflection point in the sedimentation graph were chosen.

### 2.6. Ethics

This research was considered “minimal risk”, pursuant to the resolution 8430 of 1993 of the Ministry of Health of Colombia. The study protocol was reviewed and approved by the Ethical Committee of the Faculty of Dentistry at the University of Antioquia by Act 05-2016 and carried out in accordance with the Declaration of Helsinki of 1975, revised in 2013. In addition, the study protocol was registered in Clinical Trials (NCT04658615). Data recording was used through common procedures consisting of physical, psychological, serological diagnostic exams or routine treatments. Periodontal treatment is routine therapy posing minimal risk to the patient. It does not put the researchers at risk either, since there were suitable, trained, and competent personnel to collect the information, which minimizes the physical and biological risk. Surgeries and replacement procedures for missing teeth, such as implants, were not included. Furthermore, it was based on previous scientific evidence from animal and human studies. Participants’ safety prevailed, risks were clearly expressed, and written informed consent of research subjects or their legal representative and two witnesses were obtained.

The process to obtain informed consent was as follows: The treating physician of the institutions where patients were being treated informed patients about the research and thereafter invited them to participate. Patients who accepted were informed about the research by a member of the research team and the informed consent form was given to them to read. Then, they were asked about any possible doubts, and it was confirmed that the person had understood all the information before signing the document. This process was carried out by knowledgeable and experienced professionals, qualified to take care of human being integrity. The confidentiality of all information, on the other hand, was carried out by health professionals trained for research. The necessary human and material resources to guarantee the well-being of the research subjects had to be available, and this was ensured once the authorization was obtained.

## 3. Results

A total of 217 patients meeting the condition of being seropositive for RA were preselected between March 2019 and 25 March 2020. Of those, 75 were not included because upon verifying the clinical history there were criteria that did not correspond to those of the study. The remaining 142 were evaluated by the rheumatologist in a routine consultation in the company of the research dentist in order to verify the rest of the selection criteria. Overall, 83 subjects who did not meet the dental or medical criteria did not live in the city, or refused to participate, and others who, despite having accepted, did not attend the first appointment for the intervention were excluded. A total of 59 patients were treated, out of which 7 could not be contacted for the second time, 29 had before and after measurements in all the variables proposed in the study (periodontal, blood), and 23 patients were not followed up in the blood and periodontal variables due to the effects of the worldwide SARS-CoV-2 virus pandemic (Figure 1 and Figure 2, Table 1). In order to obtain the variables that did not depend on attendance at the data collection place, given the mandatory quarantine, permission was requested from Artmedica to review their records, and the HRQoL and OHRQoL survey was applied by telephone. Out of those who received treatment, 7 patients were lost and could no longer be located.

Regarding the effect of periodontal therapy, no statistically significant differences were found in the DAS-28 before and after the procedure in the group with periodontitis and reduced periodontium, 0.2 (−0.4;0.7), *p* = 0.830 and 0.6 (−0.2;1.4), *p* = 0.167, respectively. Differences were detected after treatment in physical pain dimension in both groups, and psychological disability in the periodontitis group, 0.8 (0.2;1.4). In HRQoL, there were differences in general health in both groups. In the group with periodontitis, there were differences in physical functions, physical role, emotional role, and mental health (Table 2).

The effect of periodontal treatment was compared between the group that underwent posttest measurement of periodontal and blood parameters, and the one that, due to the pandemic, could not be evaluated (Table 3). There were statistically significant differences in the extension of the OHIP-14 impact (*p* = 0.004) and in the psychological distress dimension (*p* = 0.043). There were no statistically significant differences between the groups for SF-36 functions.

Table 4 displays the effect of the intervention in the DAS-28. It can be seen that there is a larger difference in the group that had both measurements of the blood and periodontal parameters, 0.3 (−0.1; 0.8), *p* = 0.1760, compared with the total of study subjects 5 (−0.2; 1.1), *p* = 0.106. These differences were not statistically significant.

In the principal component analysis (PCA) results, it was found that the dimensions that best explained the oral-health-related quality of life in the study population after the procedure were handicap and social disability. Meanwhile, for HRQoL, the functions best explaining HRQoL after treatment were the emotional role, mental health, and social function. These models explained the variance by 77.7% and 76.2%, respectively (Table 5).

When comparing the effect of periodontal treatment in the group of 29 patients who were followed up on blood and periodontal parameters, it was found that there were statistically significant differences before and after in psychological distress, emotional role, and mental health, which indicates an improvement in the scores of these variables (Table 6).

The multivariate linear regression model (Table 7) shows that the variables best predicting the functions of HRQoL measured with SF-36 after periodontal treatment were physical disability and average PD, considered negative predictive values of general health. Regarding the emotional role, negative predictive values were psychological discomfort, psychological disability, and physical pain. Lastly, in DAS-28, physical disability, physical pain, and the number of teeth were positive predictive values for the change in health in the last year. This means that, after periodontal treatment, for each increase in one unit of the values of these variables, the change in self-perceived health in the patients also increased. These results show statistically significant differences.

## 4. Discussion

In this 3-month quasi-experimental study, periodontal treatment had no effect on reducing disease activity in RA patients—although this indicator decreased, the differences were not statistically significant. On the other hand, differences were detected in OHIP-14 dimensions in the group with periodontitis, as well as in the health-related quality of life (HRQoL) measured with the SF-36 instrument.

These results provide new and important information in relation to the issue of quality of life and its change in RA patients treated periodontally. These results are considered to be important since it is known that RA continues to cause a modest global disability, with serious consequences in people suffering it [44,45]. Other clinical studies have also researched these effects [20]. In a randomized controlled clinical trial conducted in 22 patients, no differences in DAS-28 and HRQoL were found after periodontal treatment [46].

The results of this study gathered a wide range of biological and clinical data, which will possibly be part of future analyses comparing procedures, such as meta-analysis. This is also in good agreement with the current evidence about the effect of periodontal treatment on systemic diseases, as has been demonstrated in other studies and different systematic reviews [47,48].

A decrease in the physical pain dimension score measured with OHIP-14 after periodontal treatment and an increase in HRQoL scores could be explained because the periodontal health in patients improved significantly after they underwent postprocedure treatment 3 months later. This result appears to be biologically plausible as regards the periodontal treatment that is carried out in routine dental care and that concurs with periodontitis natural development. These results are similar to those obtained by other clinical studies [49,50].

When comparing the OHIP-14 results of the group that was followed up regarding blood and periodontal parameters with those of the group that could not be evaluated due to the pandemic lockdown, differences were found in the OHIP-14 extension and psychological discomfort. This could be explained by the lack of access to health services as a result of the lockdown caused by the health emergency in Colombia over the SARS-CoV-2 pandemic [51,52].

Several studies show changes in RA patient care due to the SARS-CoV-2 pandemic, as shown by Bonfa et al. in a paper describing that online patient care was affected by poor Internet access for some patients, a poor-quality Internet connection on both sides, a lack of easy-to-use medical files, and also patients’ psychological distress regarding online consultations, as most patients preferred face-to-face consultations with their physician [53].

According to the World Health Organization, epidemics have mental impacts on affected populations [54,55]. A study conducted in 15 Arab countries revealed an increase in negative emotions (anxiety, depression, and outrage) and a decrease in positive emotions (happiness and satisfaction) in patients with chronic rheumatic diseases [56]. Nevertheless, a study showed greater satisfaction with teleconsultation because patients did not have to travel, and considering the functional and physical limitations caused by this disease, this could lead to a better quality of life [57].

The former trends show how accessing periodontal treatment and periodic and professional check-up appointments impact people’s oral health, reflecting their impact on the dimensions and functions of HRQoL and OHRQoL. This is in line with the results of the blood and periodontal parameters and consistent with the results of other clinical trials [58,59]. A similar study conducted by Okada et al. found a statistically significant reduction (*p* < 0.001) observed in PD and PAL values in the treatment group in 26 patients 8 weeks after NSPT, compared with the control group [60].

Nguyen et al. conducted a randomized clinical trial (RCT) in 82 RA patients with periodontitis and found that DAS-28 disease activity (CRP) was significantly reduced 3 months after the procedure, both in the group treated with NSPT and in the control group (dental hygiene only). In addition, this study showed a significant decrease in ESR, ACPAs, and DAS-28-PCR in the group treated with NSPT 6 months later, while the control group showed a decrease only in ACPAs [59]. This suggests that prolonged follow-up of patients could show significant changes in study variables. Non-surgical periodontal treatment can significantly reduce DAS-28-CRP, classification of disease activity, ESR, and serum ACPA level, and it can also be applied to reduce RA severity in RA patients with periodontitis [59]. By observation, a meta-analysis analyzed the effect of non-surgical periodontal treatment in RA and non-RA patients with periodontitis, concluding that treatment is equally effective in both groups and thus suggesting that RA does not affect the clinical efficacy of non-surgical periodontal therapy [61].

Results of different investigations analyze the possible modification of drugs for RA treatment and its effect on periodontal indicators. This study’s BOP results could be explained by the fact that a quarter of the patients were being treated with biological DMARDs. A study measuring the effect of biological and non-biological drugs concluded that antirheumatic therapy (MTX and anti-TNF) has a negligible influence on the periodontal status of RA patients [50].

Few studies have been conducted with a similar aim, as this trial is the first to research the effect of periodontal treatment on the quality of life of RA patients with the use of SF-36 to assess HRQoL and the impact on oral health. It highlights the subject’s perception and can determine functional problems, discomfort, and pain. In a previous study in RA patients from the same institution (Artmedica), quality of life was worse in the emotional role and mental health, compared with the change displayed by those same functions in our study [62].

As for the oral-health-related quality of life, other studies show worse scores [63,64]. It has been shown that periodontitis affects health-related quality of life [65] and that non-surgical periodontal treatment can increase its scoring [66], especially in terms of functional limitation, psychological distress, and pain [67]. Those results resemble our findings in the present study.

Periodontitis and RA are complex and multifactorial chronic diseases; hence, treatment is recommended to involve both dentists and rheumatologists in order to contribute to the optimal management of the patient.

The strong point of this study is that it is one of the first studies to measure the effect of periodontal treatment on general and oral-health-related quality of life in this type of patient. Therefore, it outlines a new line to undertake further research in the area of dentistry and rheumatology.

Limitations in this study result from the fact that participants do not represent the total number of Artmedica patients and other RA patients. Patients were selected from the treating institution, which makes them have special characteristics in their clinical and treatment characteristics. The main limitation of this research lies in the small sample size available for analysis, which did not reach the levels calculated a priori. This was due to the beginning of the worldwide SARS-CoV-2 pandemic that prevented the procedure from being evaluated in some patients and new people from being recruited to be included in the sample.

## 5. Conclusions

Periodontal treatment had effects on health-related quality of life measured with SF-36 and OHIP-14 in RA patients. No effect on RA activity was observed. However, the clinical effect of periodontal treatment in RA patients provides important data to support periodontal care in patients.

Further research should elucidate social determinants of the quality of life in these patients through mixed methods, and the health promotion and educational strategies could be focused on the specific oral health needs of RA patients.

## Figures and Tables

**Table 1:** Baseline sociodemographic, habits and clinical characteristics of rheumatoid arthritis patients.

Variables	*n*	%
Sociodemographic		
Sex		
Male	8	15.4
Female	44	84.6
Age (years)		
27–59	35	67.3
60 and more	17	32.7
Years (X ± SD)	55.2	9.2
Education		
≤ Primary	19	36.5
Secondary	28	53.8
University	5	9.6
Socioeconomic status		
Low	8	15.4
Medium	40	76.9
High	4	7.7
Labor situation		
Employed	14	26.1
Unemployed	38	73.1
Habits. Medical history and medications		
Smoking (Yes)	2	3.8
Practice exercise (Yes)	21	40.4
Diabetes (Yes)	4	7.7
Arterial hypertension (Yes)	15	28.8
Osteoporosis (Yes)	11	21.2
NSAIDs (Yes)	14	26.9
Corticosteroids	36	69.2
Biological DMARDs	11	21.2
Non- Biological DMARDs	45	86.5
RA clinical variables		
ACPAs (Me-IQR)	83.2	10.7–325.8
RF (Me-IQR)	77.7	25.0–187.7
CPR (Me-IQR)	2.3	0.6–7.9
RA Duration in years (Me-IQR)	8.2	4.1–15.0
Type of RA		
Polyarticular	47	90.4
Oligoarticular	4	7.7
Monoarticular	1	1.9
Remission (Yes)	27	51.9
Erosive RA (Yes)	20	38.5
Self-perception variables		
Swelling (Yes)	38	73.1
Pain (Yes)	39	75.0
Morning stiffness (Yes)	37	71.2
Fatigue and depression (Yes)	29	55.8
Family support (Yes)	52	100.0
Periodontal variables		
Number of teeth in mouth (Me-IQR)	24.0	20.2–26.0
Reduced periodontium (Yes) (*n*%)	21	40.4
Periodontitis (Yes)	31	59.6
Stage of periodontal disease		
I	0	0.0
II	1	3.2
III	18	58.1
IV	12	38.7
*Porphyromonas gingivalis* (Yes)	4	7.7
PAL (Me-IQR)	2.9	1.9–3.4
PD (Me-IQR)	2.5	2.1–3.4
BoP (Me-IQR)	15.6	10.8–26.2

NSAIDS, non-steroidal anti-inflammatory drugs; DMARDS, disease-modifying antirheumatic drugs; RA, rheumatoid arthritis; ACPAs, anti-citrullinated protein/peptide antibody; Me-IQR, median- interquartile range; RF, rheumatoid factor; CPR, C-reactive protein; PAL, periodontal attachment level; PD, probing depth; BoP, bleeding on probing.

**Table 2:** Effect of periodontal treatment on DAS-28, HRQoL, and OHRQoL in patients with and without periodontitis.

Variables	Before		After		Difference (_95%_CI)	p *
	(X ± SD)	Me (IQR)	(X ± SD)	Me (IQR)		
Periodontitis (*n* = 31)						
RA						
Disease activity score-28 (DAS-28)	2.6 (1.7)	2.3 (1.4;3.2)	2.4 (1.5)	2.2 (1.6;3.0)	0.2 (−0.4;0.7)	0.830
Oral-health-related quality of life (OHRQoL)						
OHIP-14 Score	10.5 (12.0)	8.0 (0.0;14.0)	7.3 (8.5)	5.0 (0.0;10.0)	3.1 (0.1;6.1)	0.054
Extent	1.9 (3.5)	0.0 (0.0;2.0)	0.9 (2.1)	0.0 (0.0;0.0)	0.9 (−0.1;1.9)	0.065
Functional limitation	0.9 (2.1)	0.0 (0.0;0.0)	0.7 (1.2)	0.0 (0.0;1.0)	0.2 (−0.4;0.9)	0.644
Physical pain	2.5 (2.7)	2.0 (0.0;4.0)	1.2 (1.6)	0.0 (0.0;1.0)	1.2 (0.5;1.9)	0.004
Psychological discomfort	1.9 (2.5)	1.0 (0.0;4.0)	1.9 (2.1)	2.0 (0.0;4.0)	0.0 (−0.6;0.6)	0.896
Physical disability	1.8 (2.2)	0.0 (0.0:4.0)	1.2 (1.9)	0.0 (0.0;2.0)	0.5 (−0.1;1.2)	0.101
Psychological disability	1.9 (2.1)	2.0 (0.0;3.0)	1.0 (1.5)	0.0 (0.0;2.0)	0.8 (0.2;1.4)	0.015
Social disability	0.5 (1.4)	0.0 (0.0;0.0)	0.3 (0.9)	0.0 (0.0;0.0)	0.2 (−0.3;0.7)	0.593
Handicap	0.9 (1.6)	0.0 (0.0;2.0)	0.8 (1.4)	0.0 (0.0;2.0)	0.1 (−0.4;0.5)	0.721
Health-related quality of life (HRQoL)						
General health perceptions	55.3 (19.0)	52.0 (40.0;70.0)	62.3 (20.8)	62.0 (50.0;77.0)	−6.9 (−13.9;0.0)	0.037
Physical functioning	55.8 (27.3)	60.0 (30.0;80.0)	63.1 (22.6)	65.0 (45.0;85.0)	−7.2 (−15.8;1.3)	0.047
Role limitations (physical)	41.9 (45.4)	25.0 (0.0;100.0)	64.5 (43.2)	100.0 (0.0;100.0)	−22.6 (−40.7;−4.5)	0.020
Role limitations (emotional)	49.5 (50.1)	33.3 (0.0;100.0)	74.2 (42.7)	100.0 (33.3;100.0)	−24.7 (−44.9;−0.5)	0.014
Social functioning	66.9 (22.0)	33.3 (0.0;100.0)	70.2 (42.8)	100.0 (33.3;100.0)	−3.2 (−11.3;4.9)	0.459
Bodily pain	53.1 (12.4)	54.0 (50.0;62.0)	50.6 (13.8)	50.0 (42.0;62.0)	2.4 (−2.4;7.7)	0.262
Energy/vitality	55.6 (19.4)	55.0 (40.0;65.0)	60.0 (21.3)	55.0 (50.0;70.0)	−4.3 (−10.0;1.3)	0.089
Mental Health	63.5 (19.6)	56.0 (48.0;76.0)	75.6 (18.8)	76.0 (60.0;92.0)	−12.1 (−18.7;−5.5)	0.001
Health change	27.4 (29.1)	56.0 (48.0;76.0)	26.6 (28.1)	25.0 (0.0;50.0)	0.8 (−12.2;−5.5)	0.744
**Variables**	**Before**		**After**		**Difference (_95%_CI)**	p *
	**(X ± SD)**	**Me (IQR)**	**(X ± SD)**	**Me (IQR)**		
Reduced Periodontium (*n* = 21)						
RA						
Disease activity score-28 (DAS-28)	3.9 (1.7)	3.7 (2.2;5.3)	3.2 (2.0)	2.6 (2.0;4.9)	0.6 (−0.2;1.4)	0.167
Oral-health-related quality of life (OHRQoL)						
OHIP-14 Score	9.7 10.4)	4.0 (1.5;20.5)	13.0 (11.2)	10.0 (3.5;17.5)	−3.2 (−8.1;1.6)	0.196
Extent	1.6 (2.7)	0.0 (0.0;4.5)	1.9 (3.5)	0.0 (0.0;2.0)	−0.3 (−1.8;1.1)	0.623
Functional limitation	0.9 (1.4)	0.0 (0.0;2.0)	1.5 (1.5)	0.0 (0.0;2.0)	−0.1 (−0.7;0.5)	0.589
Physical pain	1.9 (2.1)	1.1 (0.0;4.0)	3.0 (1.9)	3.0 (2.0;4.0)	−1.1 (−2.0;−0.2)	0.013
Psychological discomfort	2.5 (2.2)	2.0 (0.5;4.0)	2.9 (2.4)	2.0 (1.0;4.0)	−0.3 (−1.4;0.8)	0.782
Physical disability	1.6 (2.5)	0.0 (0.0;3.0)	2.2 (2.6)	2.0 (0.0;4.0)	−0.6 (−1.9;0.6)	0.365
Psychological disability	1.4 (1.9)	0.0 (0.0;3.0)	1.9 (2.0)	2.0 (0.0;3.0)	−0.5 (−1.4;0.4)	0.235
Social disability	0.4 (0.9)	0.0 (0.0;0.5)	0.6 (1.1)	0.0 (0.0;1.0)	−0.2 (−0.6;0.2)	0.414
Handicap	0.9 (1.7)	0.0 (0.0;2.0)	1.2 (2.0)	0.0 (0.0;2.0)	−0.3 (−1.2;0.6)	0.343
Health-related quality of life (HRQoL)						
General health perceptions	53.0 (18.3)	47.0 (36.0;72.0)	46.4 (22.6)	50.0 (30.0;59.5)	6.6 (0.1;13.1)	0.046
Physical functioning	53.1 (22.5)	55.0 (37.5;75.0)	52.4 (28.3)	55.0 (22.5;77.5)	0.7 (−10.7;12.2)	0.297
Role limitations (physical)	38.1 (45.1)	0.0 (0.0;100.0)	33.3 (44.9)	0.0 (0.0;100.0)	4.7 (−1.1;25.6)	0.676
Role limitations (emotional)	52.4 (49.0)	66.7 (0.0;100.0)	57.1 (48.5)	100.0 (0.0;100.0)	−4.8 (−16.1;25.6)	0.518
Social functioning	71.4 (22.1)	75.0 (50.0;87.5)	61.3 (27.9)	62.5 (43.7;87.5)	10.2 (−1.1:21.3)	0.067
Bodily pain	46.0 (14.7)	52.0 (42.0;52.0)	51.6 (12.0)	52.0 (50.5;58.0)	5.6 (−11.4;0.2)	0.064
Energy/vitality	53.6 (13.8)	50.0 (45.0;65.0)	51.7 (18.5)	50.0 (37.5;67.5)	1.9 (4.5;8.2)	0.518
Mental Health	66.1 (15.4)	64.0 (54.0;78.0)	65.3 (16.6)	60.0 (56.0;76.0)	0.8 (−5.3;6.8)	0.759
Health change	27.4 (33.4)	25.0 (0.0;50.0)	39.3 (34.9)	50.0 (0.0;75.0)	−11.9 (−26.6;2.7)	0.128

* Wilcoxon test for repeated samples.

**Table 3:** Comparison of the effect changes according to the follow-up of patients with rheumatoid arthritis.

Variables	Followed Patients (*n* = 29)		Unfollowed Patients (*n* = 23)		Difference (_95%_CI)	p *
	(X ± SD)	Me (IQR)	(X ± SD)	Me (IQR)		
RA						
DAS-28	2.6 (1.7)	2.3 (1.7;3.1)	3.0 (1.9)	2.4 (1.7;4.2)	0.4 (−0.6;1.4)	0.562
Oral-health-related quality of life (OHRQoL)						
OHIP-14 Score	12.2 (11.5)	10.0 (3.0;17.0)	6.6 (6.8)	5.0 (0.0;10.5)	5.6 (−11.3;0.0)	0.065
Extent	2.1 (3.3)	0.0 (0.0;2.5)	0.3 (1.3)	0.0 (0.0;0.0)	−1.8 (−3.3;0.2)	0.004
Functional limitation	0.9 (1.6)	0.0 (0.0;1.5)	0.8 (1.0)	0.0 (0.0;1.5)	−0.1 (−0.8;0.7)	0.555
Physical pain	2.3 (1.9)	2.0 (0.0;4.0)	1.7 (1.8)	2.0 (0.0;3.0)	−0.6 (−1.8;0.4)	0.197
Psychological discomfort	2.9 (1.9)	2.0 (0.0;4.0)	1.6 (1.8)	1.0 (0.0;2.5)	−1.3 (−2.3;0.1)	0.043
Physical disability	2.3 (2.6)	1.0 (0.0;5.0)	0.9 (1.1)	0.0 (0.0;2.0)	−1.4 (−2.6;0.2)	0.117
Psychological disability	1.8 (2.0)	1.0 (0.0;5.0)	0.9 (1.1)	0.0 (0.0;2.0)	−0.9 (−1.9;0.6)	0.124
Social disability	0.6 (1.3)	0.0 (0.0;0.5)	0.3 (0.7)	0.0(0.0;0.0)	−0.3 (−0.9;0.2)	0.353
Handicap	1.3 (2.0)	0.0 (0.0;2.0)	0.5 (0.9)	0.0 (0.0;1.0)	−0.8 (−0.9;0.2)	0.175
Health-related quality of life (HRQoL)						
General health perceptions	55.1 (26.3)	55.0 (37.5;78.5)	55.8 (17.5)	57.0 (42.5;71.0)	0.7 (−12.6;13.9)	0.945
Physical functioning	56.2 (25.5)	60.0 (35.0;75.0)	59.3 (24.9)	60.0 (37.5;80.0)	−3.1 (−11.5;17.6)	0.629
Role limitations (physical)	42.2 (43.9)	25.0 (0.0;100.0)	60.7 (47.8)	100.0 (0.0;100.0)	−18.5 (−7.7;44.7)	0.132
Role limitations (emotional)	66.7 (45.4)	100.0 (0.0;100.0)	65.1 (47.7)	100.0 (0.0;100.0)	−1.6(−28.3;25.1)	0.891
Social functioning	68.1 (31.0)	62.5 (37.5;100.0)	61.9 (21.1)	62.5 (50.0;75.0)	−6.2 (−22.0;9.6)	0.287
Bodily pain	53.0 (11.3)	51.0 (43.0;58.0)	49.3 (14.9)	51.0 (42.0;62.0)	−3.7 (−11.1;3.7)	0.539
Energy/vitality	58.9 (23.4)	55.0 (42.5;80.0)	51.0 (12.6)	50.0 (42.5;57.5)	−8.0 (−19.3;3.2)	0.240
Mental Health	74.5 (20.2)	76.0 (60.0;96.0)	65.3 (14.3)	64.0 (54.0;76.0)	−9.1 (−19.5;1.2)	0.088
Health change	35.3 (31.0)	25.0 (0.0;50.0)	29.8 (32.2)	25.0 (0.0;50.0)	5.1 (−23.7;12.6)	0.457

_95%_CI: 95% confidence interval; * U Mann–Whitney test for independent samples.

**Table 4:** Effect of the periodontal procedure on rheumatoid arthritis activity.

Variables	Before		After		Difference (_95%_CI)	p *
	(X ± SD)	Me (IQR)	(X ± SD)	Me (IQR)		
RA						
DAS-28 (*n* = 52)	3.1 (1.8)	2.3 (1.9;4.7)	2.7 (1.8)	2.2 (1.4;3.4)	0.3 (−0.1;0.8)	0.176
DAS-28 (*n* = 29)	3.1 (1.7)	2.7 (2.0;4.3)	2.6 (1.7)	2.2 (1.7;3.1)	0.5 (−0.2;1.1)	0.106

_95%_CI: 95% confidence interval; * Wilcoxon test for repeated samples.

**Table 5:** Factor analysis of OHIP-14 and SF-36 before and after periodontal treatment (*n* = 52).

Variables	Before					After				
	Components		KMO and Bartlett’s Test	*p*	Explained Variance (%)	Components		KMO and Bartlett’s Test	*p*	Explained Variance (%)
	1	2				1	2			
Oral-health-related quality of life (OHRQoL)										
Functional limitation	0.843	0.285	0.870	0.000	79.2	0.452	0.615	0.872	0.000	77.7
Psychological discomfort	0.588	0.643				0.694	0.499			
Physical disability	0.401	0.821				0.609	0.586			
Psychological disability	0.752	0.535				0.628	0.630			
Social disability	0.804	0.204				0.866	0.307			
Handicap	0.894	0.246				0.918	0.183			
Health-related quality of life (HRQoL)										
General health perceptions	0.578	0.446	0.827	0.000	73.4	0.404	0.792	0.880	0.000	76.2
Role limitations (emotional)	0.525	0.593				0.883	0.156			
Social functioning	0.844	0.186				0.763	0.417			
Energy/vitality	0.831	0.351				0.640	0.562			
Mental Health	0.839	0.293				0.845	0.383			

Estimation and extraction method: component analysis. Rotation method: Varimax with Kaiser’s normalization.

**Table 6:** Effect of periodontal treatment on HRQoL and OHRQoL in rheumatoid arthritis patients.

Variables	Before		After		Difference (_95%_CI))	p *
	(X ± SD)	Me (IQR)	(X ± SD)	Me (IQR)		
Oral-health-related quality of life (OHRQoL)						
OHIP-14 Score	12.4 (11.5)	4.0 (0.5–17.5)	12.2 (11.5)	10.0 (3.0–17.0)	−1.9 (−5.9;2.0)	0.230
Extent	1.9 (3.6)	0.0 (0.0–3.0)	2.1 (3.4)	0.0 (0.0–2.5)	−0.2 (−1.4;1.0)	0.511
Functional limitation	1.2 (2.1)	0.0 (0.0–2.0)	0.9 (1.6)	0.0 (0.0–1.5)	0.3 (−0.3;0.9)	0.359
Physical pain	2.1 (2.4)	1.0 (0.0–4.0)	2.3 (1.9)	2.0 (0.0–4.0)	−0.3 (−1.3;0.7)	0.440
Psychological discomfort	2.1 (2.4)	2.0 (0.0–4.0)	3.0 (2.4)	2.0 (0.5–5.5)	−0.9 (−1.7;−0.1)	0.037
Physical disability	1.8 (2.5)	0.0 (0.0–3.5)	2.3 (2.6)	1.0 (0.0–5.0)	−0.5 (−1.5;0.4)	0.330
Psychological disability	1.7 (2.2)	1.0 (0.0–3.0)	1.8 (2.0)	1.0 (0.0–3.0)	−0.1 (−0.9;0.8)	0.867
Social disability	0.6 (1.5)	0.0 (0.0–0.5)	0.6 (1.2)	0.0 (0.0–0.5)	0.0 (−0.6;0.1)	0.786
Handicap	0.8 (1.6)	0.0 (0.0–1.0)	1.3 (2.0)	0.0 (0.0–2.0)	−0.5 (−1.1;1.1)	0.151
Health-related quality of life (HRQoL)						
General health perceptions	51.9 (18.3)	47.0 (36.0–69.5)	55.1 (26.3)	55.0 (37.5–78.5)	−3.1 (−10.7;4.4)	0.301
Physical functioning	51.7 (24.5)	60.0 (35.0–75.0)	56.2 (25.5)	60.0 (35.0–75.0)	−4.4 (−14.4;5.4)	0.607
Role limitations (physical)	37.9 (42.6)	25.0 (0.0;87.5)	42.2 (43.9)	25.0 (0.0–100.0)	−28.7 (−47.1;−10.4)	0.545
Role limitations (emotional)	50.6 (49.3)	33.3 (0.0–100.0)	66.7 (45.2)	100.0 (0.0–100.0)	−16.1 (−34.8;2.7)	0.046
Social functioning	69.8 (20.7)	75.0 (50.0–87.5)	68.1 (31.2)	62.5 (37.5–100.0)	−1.7 (−9.2;12.7)	0.644
Bodily pain	50.2 (13.2)	52.0 (42.0–57.0)	53.0 (11.3)	51.0 (43.0–58.0)	−2.8 (−9.4;3.7)	0.315
Energy/vitality	56.9 (17.2)	55.0 (45.0–70.0)	59.0 (23.4)	55.0 (42.5–80.0)	−2.0 (−8.0;3.9)	0.653
Mental Health	64.5 (18.9)	56.0 (50.0–78.0)	74.5 (20.1)	76.0 (60.0–96.0)	−9.9 (−17.5;−2.4)	0.019
Health change	31.0 (33.2)	25.0 (0.0–50.0)	35.3 (30.9)	25.0 (0.0–50.0)	−4.3 (−17.5;8.9)	0.530

* Wilcoxon test for repeated samples.

**Table 7:** Linear regression model for dimensions with significant correlations with OHIP-14 and periodontal variables.

Variables with More Than Four Significant Correlations According to Spearman’s Rho (*n* = 29)	Variables in the Model	Before		After	
		Regression Coefficient	Determination Coefficient (%)	Regression Coefficient	Determination Coefficient (%)
**Health-Related Quality of Life (HRQoL)**					
General health perceptions	DAS-28	0.112	20.6	−0.123	5.7
	Physical disability	−0.001		−0.103 *	
	Psychological disability	−0.700 *		−0.107	
	Physical pain	−0.595 *		−0.164	
	PD Mean	−0.149		−0.310 *	
Role limitations (emotional)	DAS-28	−0.015	8.7	−0.305	12.2
	Physical disability	0.121		0.19	
	Psychological discomfort	−0.329 *		−0.358 *	
	Psychological disability	−0.326 *		−0.380 *	
	Physical pain	0.104		−0.262 *	
	PD Mean	0.253		0.154	
Health change	DAS-28	0.076	18.2	0.348 *	18.0
	Physical disability	−0.291 *		0.349 **	
	Psychological disability	0.535		−0.118	
	Physical pain	0.085		0.323 *	
	PD Mean	−0.264		0.001	
	Number of teeth in mouth	0.314 *		0.327 *	

* *p* ≤ 0.05, ** *p* < 0.01.

## Data Availability

The data that support the findings of this study are available from the corresponding author, upon reasonable request.
